# Amino Acid Balanced Compound Low-Protein Diets Improve Resource Efficiency in Sanhua Goose Production: Impacts on Metabolism, Gut Health, and Microbial Diversity

**DOI:** 10.3390/microorganisms13092179

**Published:** 2025-09-18

**Authors:** Xianze Wang, Huiying Wang, Yi Liu, Guangquan Li, Daqian He, Shufang Chen, Huiyan Jia, Jiuli Dai, Xiao Zhou

**Affiliations:** 1 Institute of Animal Husbandry and Veterinary Medicine, Shanghai Academy of Agricultural Science, Shanghai 201106, China; wxz13187058389@163.com (X.W.); wanghy2010@189.cn (H.W.); liuyi20031194@163.com (Y.L.); lgqdx123@126.com (G.L.); 2 Institute of Livestock and Poultry Research, Ningbo Academy of Agricultural Sciences, Ningbo 315040, China; 13606780161@163.com (S.C.); jhynku@163.com (H.J.); 13858357201@163.com (J.D.); xiaozhou_2023@163.com (X.Z.)

**Keywords:** growth performance, limiting amino acid balancing, soybean meal reduction, intestinal morphology, cecal microbiota

## Abstract

This study investigated a compound low-protein diet (CLPD) strategy to reduce soybean meal (SBM) dependency in meat geese. Diets were formulated with crude protein (CP) levels decreasing from 16.5% (corn-soybean meal diet, CSD) to 9.8%, incorporating alternative ingredients such as rapeseed meal, corn distillers dried grains with solubles (DDGS), broken rice, and rice bran. All diets were balanced for limiting amino acids (lysine, methionine, threonine, and valine) through supplemental synthetic amino acids. A total of 192 four-week-old Sanhua geese were randomly assigned according to a single-factor completely randomized design to four dietary treatment groups: the 16.5% (CSD) group and three CLPD treatment groups (14.0% CP, 11.5% CP, and 9.8% CP). Each treatment consisted of six replicate pens with eight geese per pen. During the six-week trial, evaluations included growth performance, organ weights, nutrient digestibility, serum biochemistry, amino acid profiles, intestinal morphology, and cecal microbiota composition. Results demonstrated that compared to the 16.5% (CSD) group, the 11.5% CP (CLPD) group significantly improved final body weight (*p* < 0.05), average daily gain (P_Linear < 0.01, *p* < 0.05), and feed conversion efficiency (P_Linear < 0.01, *p* < 0.05), alongside enhanced apparent digestibility of crude protein and amino acids (P_Linear < 0.01, *p* < 0.05). Organ weights were generally stable, though the 9.8% CP (CLPD) group showed reduced liver weight (*p* < 0.05) and increased abdominal fat (P_Linear < 0.01, *p* < 0.05). Serum levels of low-density lipoprotein cholesterol increased (P_Linear < 0.05, *p* < 0.05). Intestinal morphology improved in the duodenum and jejunum: in the duodenum, villus height and villus-to-crypt ratio were significantly increased, and crypt depth was significantly decreased (P_Linear < 0.01, *p* < 0.05); in the jejunum, villus height was significantly increased (*p* < 0.05) and crypt depth was significantly decreased (*p* < 0.05). Cecal microbiota alpha diversity remained consistent. The dominant genera in the 9.8% CP (CLPD) group were *unclassified_Oscillospiraceae* and *unclassified_Ruminococcaceae* (*p* < 0.05), among which, *Megamonas*, *Prevotellaceae_Ga6A1_group*, and *Rikenellaceae_RC9_gut_group* dominated in the 16.5% (CSD) group (*p* < 0.05). These findings indicate that a compound low-protein diet (CLPD) with 11.5% CP, precisely balanced for limiting amino acids, supports optimal growth performance, improves nutrient utilization, and maintains intestinal health in meat geese. Overall, this offers a viable approach to easing SBM reliance in poultry nutrition while enhancing resource efficiency.

## 1. Introduction

SBM, with its high crude protein content (40–48%) and balanced amino acid profile, serves as the primary protein source in poultry feed, contributing approximately 60% of dietary protein [1]. However, the rapid intensification of livestock farming has driven surging global soybean demand, increasing Chicago Board of Trade (CBOT) soybean futures prices by USD 9.47 per bushel during 2020–2023, thereby significantly reducing profit margins in animal production. This supply-demand imbalance highlights three systemic challenges: (1) Geopolitical Concentration: The United States and Brazil collectively dominate 60% of global soybean production, 90% of which comprises genetically modified varieties [2]. Meanwhile, China’s import dependency exceeds 80%, exposing its industry to trade volatility, such as tariff disputes that inflate costs; (2) Ecological Pressures: Global soybean cultivation areas expanded by 108% from 2000 to 2019, directly linked to 9% of South American deforestation [3]. The overall average carbon footprint of Brazilian soybean exports is 0.69 tons of carbon dioxide equivalent per ton of soy equivalent [4], while excessive SBM use contributes to 41.6% of livestock nitrogen emissions [5]; (3) Resource Constraints: A 10% reduction in SBM usage could conserve 11.3 million hectares of arable land annually (equivalent to Bulgaria’s land area) and reduce nitrogen emissions by 1.4 million tons [6]. These challenges underscore the urgent need for sustainable SBM reduction strategies to ensure food security and ecological preservation.

Low-protein diets (LPD) based on the ideal amino acid model and supplemented with synthetic amino acids offer a viable strategy for conserving protein resources. Alterations in dietary composition, particularly protein levels, can influence the gut microbiota structure, thereby modulating nutrient utilization efficiency and environmental outcomes [7,8]. While LPD has demonstrated potential to reduce nitrogen emissions in broilers [9], its application in goose nutrition remains largely underexplored. Notably, global goose production reached 770 million birds in 2023, with China contributing over 97% of this total and an annual growth rate of approximately 1% [10]. Despite this, research on SBM reduction in geese constitutes less than 3% of total poultry-related studies. As herbivorous poultry, geese exhibit unique digestive adaptations, such as robust cecal fermentation capacity, which may confer greater flexibility in adapting to dietary protein adjustments. Experimental evidence indicates that reducing crude protein levels in gosling diets from 18.5% to 15.5%, while balancing essential amino acids, decreases nitrogen excretion by 23% without compromising growth [11]. More recent findings suggest that lowering dietary protein and supplementing key amino acids during the gosling phase not only sustains growth performance into the growing period but also consistently reduces nitrogen excretion [12]. Additionally, prioritizing supplementation of sulfur-containing amino acids, such as methionine, at consistent protein levels has been shown to enhance slaughter rate and muscle quality in geese [13].

Despite these advances, research on low-protein, amino acid-balanced diets in geese has predominantly focused on goslings and breeding geese [14], with systematic studies on meat geese (commercial geese) remaining scarce. Meat geese, characterized by a prolonged rearing period and limited suitability for restricted feeding, may exhibit heightened adaptability to LPD [15], underscoring the practical significance of investigating SBM reduction strategies in this population. However, current studies primarily emphasize single SBM reduction paired with comprehensive amino acid balancing or supplementation with a single amino acid [16]. While these approaches confirm the feasibility of LPD in goose production, incorporating major grain processing by-products—such as DDGS, broken rice, rice bran, and wheat bran—alongside SBM reduction could diversify dietary composition, substantially lower costs, and enhance resource efficiency. Moreover, precise balancing of limiting amino acids in LPD holds promise for elucidating the amino acid metabolic requirements and nutrient deposition patterns in geese, providing a robust theoretical foundation for future optimization of meat goose diets.

The Sanhua Goose is an excellent meat goose breed widely raised across China, boasting both outstanding growth performance and strong stress resistance. According to the recommendations of the Chinese agricultural industry standards “Nutritional Requirements for Geese” (NY/T 4641-2025 [17]) and “Feeding Standards for Commercial Meat Geese” (DB37/T 2784-2016 [18]), the nutritional needs for growing geese aged 4–10 weeks include a dietary metabolizable energy of 11.0–11.5 MJ/kg, a CP level of approximately 14–16%, lysine (0.75–0.9%), methionine + cystine (0.64–0.65%), threonine (0.40–0.60%), and valine (0.61–0.67%) to support normal growth. Building on this, the present study employs a gradient SBM replacement strategy, based on the metabolizable energy levels and apparent digestibility of amino acids in geese for alternative ingredients such as rapeseed meal, DDGS, broken rice, and rice bran. By supplementing crystalline amino acids to achieve balance in key limiting amino acids, a CLPD for meat geese was formulated. Through systematic assessment of its effects on growth performance, nitrogen utilization efficiency, and intestinal development, combined with 16S rRNA gene sequencing to characterize shifts in gut microbiota structure, this research seeks to clarify the nutritional metabolic profiles of geese under reduced SBM conditions and the interplay between amino acid balance and gut microbiota. These insights aim to provide scientific guidance for advancing sustainable waterfowl production.

## 2. Research Methods and Materials

### 2.1. Animal Ethics Guidelines

The experimental protocol for this study received approval from the Shanghai Academy of Agricultural Sciences’ Animal Care and Use Committee (SAASPZ0522050, 1 April 2022). It was conducted in compliance with China’s national standards, including the ’Experimental Animal—Guidelines for Welfare’ (GB/T 42011-2022 [19]). All procedures were designed to minimize distress and discomfort for the geese throughout the study.

### 2.2. Experimental Animals and Diet Design

A total of 192 healthy 4-week-old Sanhua geese were sourced from the Daluyuan Breeding Cooperative in Lu’an City, Anhui Province, China. After a 3-day acclimatization period, the geese were weighed to determine their initial body weights. They were then randomly assigned to one of four dietary treatment groups based on these initial weights: a control group fed a Corn-Soybean meal Diet (CSD) with 16.5% crude protein (CP) and three Composite Low-Protein Diet (CLPD) groups with 14.0% CP, 11.5% CP, and 9.8% CP, respectively. Each group comprised six replicates, with eight geese per replicate. The CLPDs were formulated to match the amino acid profile of the CSD, with synthetic amino acids added to achieve this balance. To maintain energy adequacy while reducing crude protein levels, the protein-to-energy ratios were adjusted as follows: 14.86 g CP/MJ ME for the 16.5% CP (CSD) group (control), 12.62 g CP/MJ ME for the 14.0% CP (CLPD) group, 10.37 g CP/MJ ME for the 11.5% CP (CLPD) group, and 8.84 g CP/MJ ME for the 9.8% CP (CLPD) group. All dietary formulations adhered to the Nutritional Requirements for Geese in China (NY/T 4641-2025) and the Nutritional Standards for Commercial Meat Geese (DB37/T 2784-2016).

### 2.3. Feeding Management and Facility Conditions

The experiment was conducted over 6 weeks at the Zhuanghang Comprehensive Experimental Station, Shanghai Academy of Agricultural Sciences, Shanghai, China. Geese were housed in an environmentally controlled facility, with temperatures maintained between 22 and 28 °C and relative humidity ranging from 55 to 65%. Twenty-four raised mesh pens (dimensions: 3.0 m × 2.0 m × 0.8 m) were used, each fitted with plastic mesh flooring featuring 1 cm apertures. Each pen was equipped with trough feeders for ad libitum feed access and nipple drinkers for water supply. Standardized biosecurity measures were enforced, including vaccinations against Newcastle disease and avian influenza, weekly removal of pen waste, and daily monitoring of goose health status.

### 2.4. Nutrient Composition Determination

The CP content of feed and fecal samples was determined according to the method described in AOAC 984.13 (AOAC, 2005 [20]). The amino acid profiles were analyzed by Shanghai Kailite Agricultural Product Testing Technology Service Co., Ltd. (Shanghai, China) following the method specified in AOAC Method 994.12 (AOAC, 2019 [21]). The detailed breakdown of nutrient components in the feed of each treatment group is shown in Table 1.

### 2.5. Growth Performance Evaluation

On the day the experiment commenced (4 weeks of age), geese were subjected to a 12-h fasting period prior to weighing, and their initial body weight (IBW) was recorded. On the final day of the experiment (10 weeks of age), the same fasting procedure was applied before weighing to determine their final body weight (FBW), from which the average daily gain (ADG) was calculated. Throughout the experimental period, feed consumption was monitored for each replicate pen, and the feed-to-gain ratio (F/G) was determined for each replicate. The average daily feed intake (ADFI) for each replicate was subsequently calculated based on the F/G and ADG values.

The following formulas were used to assess growth performance parameters:

ADG = (FBW− IBW)/Number of Experimental Days

F/G = Total Feed Consumption/Total Weight Gain

ADFI = total feed intake/(Number of Experimental Days × number of experimental geese)

### 2.6. Relative Organ Weight Determination

On the final day of the experiment, eight geese with similar body weights were selected from each treatment group, totaling 32 geese. Specifically, 1 representative goose (matching the average body weight of the pen) was selected from each of the 6 pens (replicates) in the treatment group, and an additional 2 geese were supplementarily selected from the same treatment group to ensure consistency with the overall average body weight of the group. After an 8-h fasting period, the geese were anesthetized through a wing vein injection of 30 mg/kg sodium pentobarbital (Sinopharm Group, Shanghai, China). Once fully anesthetized, they were euthanized by exsanguination via the jugular vein, following ethical guidelines for animal welfare. The abdominal cavity was then opened, and the heart, liver, spleen, gizzard, and abdominal fat pad were carefully dissected and weighed. All organ weights were recorded by a single technician to minimize measurement variation. Relative organ weight was calculated using the formula:

Relative Organ Weight = (Organ Weight/Body Weight) × 100%

### 2.7. Determination of Apparent Digestibility Nutrient

Representative feed samples were collected from each treatment group throughout the trial to accurately reflect the nutritional composition of the diets consumed by the geese. For excreta collection, one goose per replicate pen, selected based on body weight closest to the group mean (total of 6 geese per treatment group), was used. These geese were acclimated to individual metabolic cages for a 3-day adaptation period, during which they received the experimental diet ad libitum to facilitate environmental adjustment and minimize stress-induced variability in excretion patterns. Following adaptation, excreta were collected continuously and completely over a 48-h period. Any feathers or spilled feed mixed in the excreta were carefully removed, and the samples were treated with 10% hydrochloric acid for nitrogen fixation to prevent ammonia volatilization. The treated excreta were immediately stored at −20 °C to inhibit microbial degradation and nutrient loss. For analysis, both feed and excreta samples were thawed, homogenized, and dried at 65 °C in a forced-air oven to constant weight (72 h). The dried samples were then ground through a 1-mm sieve using a laboratory mill and stored in airtight containers at room temperature until further analysis. Crude protein content was determined using the Kjeldahl method according to AOAC 984.13 (AOAC, 2005 [20]), and amino acid profiles were analyzed via ion-exchange chromatography following AOAC 994.12 (AOAC, 2019 [21]). The acid-insoluble ash (AIA) content was determined following the GB/T 23742-2009 standard [22] and the equivalent AOAC 975.12 method. First, the samples were ashed at 600 °C, followed by acid treatment to separate and quantify the insoluble fraction.

Apparent digestibility (AD) was calculated as:

AD (%) = 100 − [100 × (AIA_feed/AIA_feces) × (nutrient_feces/nutrient_feed)],

where AIA_feed and AIA_feces are the AIA percentages in feed and feces, respectively, and nutrient_feed and nutrient_feces are the concentrations of crude protein or specific amino acids in feed and feces, respectively.

### 2.8. Blood Biochemistry and Amino Acid Profile Analysis

On the final day of the experiment, following an 8-h fasting period, 5 mL blood samples were collected from the brachial vein of geese. The samples were allowed to clot at 4 °C for 2 h and then centrifuged at 3000 rpm for 10 min to separate the serum. The resulting serum samples were stored at −80 °C and subsequently transported to Shanghai Renjie Biotechnology Co., Ltd. (Shanghai, China) for biochemical analysis. Serum biochemical parameters, including total protein (TP), albumin (Alb), globulin (Glob), total cholesterol (TC), low-density lipoprotein cholesterol (LDL-C), high-density lipoprotein cholesterol (HDL-C), uric acid (UA), serum creatinine (SCR), glucose (Glu), total bilirubin (TBIL), indirect bilirubin (IBIL), alanine aminotransferase (ALT), aspartate aminotransferase (AST), and alkaline phosphatase (ALP), were measured using an automatic biochemical analyzer (Model 7180, Hitachi, Tokyo, Japan), with all procedures conducted in strict accordance with the manufacturer’s instructions.

### 2.9. Determination of Amino Acid Profiles in Serum and Breast-Leg Muscle Tissues

Serum samples were collected as described in Section 2.7 and stored at −80 °C until analysis. Following euthanasia, approximately 5 g of tissue was excised from the same anatomical locations in the breast and leg muscles of each goose. These samples were immediately placed in cryogenic tubes, snap-frozen in liquid nitrogen, and maintained at −80 °C until further processing. The amino acid composition of serum and muscle tissues was determined using a Hitachi automatic amino acid analyzer (Model: LA8080, Hitachi High-Tech Corporation, Tokyo, Japan), in accordance with the standards outlined in GB/T 18246-2019 [23] and AOAC Method 994.12 (AOAC, 2019). Briefly, sample pretreatment involved hydrochloric acid extraction for serum to isolate free amino acids, followed by precipitation removal and pH adjustment for purification; for muscle, proteins were first hydrolyzed with acid to release amino acids, followed by similar purification steps to eliminate impurities. Subsequently, target amino acids were separated via cation-exchange chromatography based on differences in their charge properties. Post-column derivatization with ninhydrin was performed online, and the derivatives were detected photometrically at dual wavelengths (570 nm for primary amino acids and 440 nm for secondary amino acids such as proline). Finally, amino acids were identified qualitatively by retention time and quantified using the external standard method with calibration curves to determine concentrations in serum and muscle.

### 2.10. Morphological Observation of the Small Intestine

Following euthanasia, the abdominal cavity was opened, and the duodenum, jejunum, and ileum were carefully isolated, rinsed with phosphate-buffered saline (PBS), and fixed in 4% paraformaldehyde solution. After 12 h, the fixative was replaced, and the samples were sent to Beijing Lanyi Technology Co., Ltd., Beijing, China, for histological processing. The fixed samples were subjected to gradient ethanol dehydration, paraffin embedding, sectioning at 5 μm, H&E staining, and mounting. Sections were examined using an Olympus BX-41TF microscope (Olympus Corporation, Tokyo, Japan). For each intestinal segment, six sections were analyzed, and measurements of villus height (VH), crypt depth (CD), and muscularis mucosae thickness were obtained from morphologically intact areas. The VH/CD ratio was calculated, and each parameter was measured five times across different areas of the section, with the average value being used for analysis.

### 2.11. Cecal Microbiota Analysis

During the slaughter process, cecal contents were collected from each goose. The samples were immediately transferred into cryogenic tubes and snap-frozen in liquid nitrogen, then sent to Beijing Biomarker Technologies Co., Ltd. (Beijing, China) for 16S rRNA gene sequencing. Microbial DNA was extracted from the cecal contents using the Tiangen DP328 Stool DNA Kit (Tiangen Biotech, Beijing, China). Following an assessment of DNA quality and concentration, the V3-V4 region of the bacterial 16S rRNA gene was amplified by PCR with the specific primers F (5′-ACTCCTACGGGAGGCAGCA-3′) and R (5′-GGACTACHVGGGTWTCTAAT-3′). The resulting amplicons were purified via gel electrophoresis and used to construct sequencing libraries. After quality control, paired-end sequencing was performed on the Illumina MiSeq 6000 platform. Raw sequencing data were processed using DADA2 for quality filtering, denoising, merging, and chimera removal to generate high-quality operational taxonomic units (OTUs). These OTUs were taxonomically annotated against the SILVA 138 database and analyzed to assess the diversity, composition, and relative abundance of the cecal microbiota in goslings.

## 3. Statistical Analysis

All data were preprocessed using Microsoft Excel, followed by statistical analyses performed in Python 3.12 with the SciPy, Statsmodels, and Pingouin libraries. One-way analysis of variance (ANOVA) was employed to assess intergroup differences, with Tukey’s multiple comparison test applied for post-hoc analysis when significant differences were detected by ANOVA (*p* < 0.05). To evaluate trends across the graded crude protein (CP) levels (16.5%, 14.0%, 11.5%, and 9.8%), orthogonal polynomial contrasts were applied using unequally spaced coefficients to test for linearity, with significance set at *p* < 0.05 for trend effects. Prior to ANOVA and polynomial contrasts, the Shapiro-Wilk test was used to verify the normality of data distribution, and Levene’s test was employed to evaluate variance homogeneity. To enhance statistical robustness and account for multiple comparisons, *p*-values were adjusted using the false discovery rate (FDR) method (α = 0.05) according to Benjamini and Hochberg (BH) to minimize the risk of Type I errors. Statistical significance was set at *p* < 0.05.

## 4. Result

### 4.1. Growth Performance

Table 2 presents the growth performance results. No significant differences were observed in IBW among the treatment groups (*p* > 0.05). Compared to the 16.5% CP (CSD) group, the FBW was significantly higher in the 11.5% CP (CLPD) and 9.8% CP (CLPD) groups (*p* < 0.01). The ADG in the 14.0% CP (CLPD), 11.5% CP (CLPD), and 9.8% CP (CLPD) groups was significantly superior to that in the 16.5% CP (CSD) group (*p* < 0.01). The F/G in the 16.5% CP (CSD) group was significantly higher than in the other groups (*p* < 0.01). No significant differences were observed in ADFI among the groups (*p* > 0.05). The CP levels exhibited significant linear effects on FBW, ADG, and F/G (P_linear < 0.01), indicating improved growth efficiency with reduced CP in the CLPD groups.

### 4.2. Relative Organ Weight

The effects of various dietary treatments on the relative organ weights and abdominal fat yield of geese are presented in Table 3. No significant differences were observed in the relative weights of the heart, spleen, and gizzard across all treatment groups (*p* > 0.05). In contrast, the relative liver weight in the 9.8% CP (CLPD) group was significantly lower than that in the other groups (*p* < 0.05). Furthermore, abdominal fat yield was significantly higher in the 11.5% CP (CLPD) and 9.8% CP (CLPD) compared to the 16.5% CP (CSD) and 14.0% CP (CLPD) groups (*p* < 0.01). The crude protein level exhibited a significant linear relationship with the abdominal fat percentage in Sanhua geese (P_linear < 0.01), indicating that lower dietary protein levels facilitate greater fat deposition.

### 4.3. Apparent Digestibility of Crude Protein and Amino Acids in Geese

The apparent digestibility of CP and amino acids in geese was significantly influenced by different dietary treatments, as shown in Table 4. CP digestibility was significantly higher in the 9.8% CP (CLPD) group compared to all other groups (*p* < 0.01), while the 11.5% CP (CLPD) group had significantly greater CP digestibility than the 16.5% CP (CSD) and 14.0% CP (CLPD) groups (*p* < 0.01). In terms of amino acid digestibility, the 9.8% CP (CLPD) group demonstrated significantly higher values for 13 amino acids—including threonine (Thr), serine (Ser), glutamic acid (Glu), alanine (Ala), valine (Val), isoleucine (Ile), leucine (Leu), tyrosine (Tyr), phenylalanine (Phe), lysine (Lys), histidine (His), arginine (Arg), and proline (Pro)—compared to all other groups (*p* < 0.01). The 11.5% CP (CLPD) group also exhibited significantly higher digestibility of CP and several amino acids, including aspartic acid (Asp), Thr, Ser, glycine (Gly), Ile, Leu, and Phe, compared to the 16.5% CP (CSD) and 14.0% CP (CLPD) groups (*p* < 0.01). However, the digestibility of Ala in the 11.5% CP (CLPD) group was significantly higher than in the 16.5% CP (CSD) group (*p* < 0.01) but did not differ significantly from the 14.0% CP (CLPD) group (*p* > 0.05). No significant differences (*p* > 0.05) were found in the digestibility of His, Arg, and Pro between the 11.5% CP (CLPD) group and the 16.5% CP (CSD) and 14.0% CP (CLPD) groups. The CP levels exhibited significant linear effects on the apparent digestibility of amino acids (P_linear < 0.01), indicating that reduced CP in the CLPD groups significantly improved amino acid digestibility.

### 4.4. Serum Biochemical Parameters

As shown in Table 5, different dietary treatments significantly influenced the serum biochemical parameters of geese. Low-density lipoprotein cholesterol (LDL-c) levels were significantly higher in the 9.8% CP (CLPD) group than in all other groups (*p* < 0.01), while no significant differences were found among the 16.5% CP (CSD), 14.0% CP (CLPD), and 11.5% CP (CLPD) groups (*p* > 0.05). Serum creatinine (SCR) levels in the 16.5% CP (CSD) and 14.0% CP (CLPD) groups were significantly higher than in the 11.5% CP (CLPD) and 9.8% CP (CLPD) groups (*p* < 0.01). Other serum biochemical parameters showed no significant differences among the treatment groups (*p* > 0.05).

### 4.5. Serum Amino Acid Composition

As shown in Table 6, there was no significant difference in serum amino acid levels among all treatment groups (*p* > 0.05). The serum levels of Asp, Glu, Ile, Leu, and Arg exhibited a significant linear relationship with dietary protein levels (P_linear < 0.01), indicating a linear decreasing trend in the concentrations of these amino acids in serum as CP levels were reduced in the CLPD groups.

### 4.6. Amino Acid Composition in Goose Breast Muscle

As shown in Table 7, different dietary treatments significantly affected the amino acid composition in goose breast muscle. The levels of Gly, Ala, Lys, Arg, and Pro in the 16.5% CP (CSD) and 14.0% CP (CLPD) groups were significantly higher than those in the 11.5% CP (CLPD) and 9.8% CP (CLPD) groups (*p* < 0.01), with no significant difference observed between the 11.5% CP (CLPD) and 9.8% CP (CLPD) groups (*p* > 0.05). Similarly, the levels of Ile, Leu, and His in the 16.5% CP (CSD) and 14.0% CP (CLPD) groups were significantly elevated compared to the 11.5% CP (CLPD) and 9.8% CP (CLPD) groups (*p* < 0.05), and no significant difference was found between the 11.5% CP (CLPD) and 9.8% CP (CLPD) groups (*p* > 0.05). For Tyr and Phe, a gradient pattern was observed, with the 16.5% CP (CSD) group exhibiting significantly higher levels than the 11.5% CP (CLPD) and 9.8% CP (CLPD) groups (*p* < 0.01), and the 14.0% CP (CLPD) group showing significantly higher levels than the 9.8% CP (CLPD) group (*p* < 0.01); however, no significant differences were detected between the 14.0% CP (CLPD) and 11.5% CP (CLPD) groups or between the 11.5% CP (CLPD) and 9.8% CP (CLPD) groups (*p* > 0.05). In contrast, the levels of ASP, Thr, Ser, Glu, Val, and Met showed no significant differences among all treatment groups (*p* > 0.05). With the exception of Asp, Glu, and Ala, all amino acid levels in the breast muscle exhibited a significant linear relationship with crude protein levels (P_linear < 0.05), indicating a decreasing trend in the deposition of these amino acids in the breast muscle as CP levels were reduced in the CLPD groups.

### 4.7. Amino Acid Composition in Goose Leg Muscle

As presented in Table 8, different dietary treatments significantly influenced the amino acid composition of goose leg muscle. The results indicated that the levels of Tyr and Phe in the 9.8% CP (CLPD) group were significantly higher than those in the 16.5% CP (CSD) and 14.0% CP (CLPD) groups (*p* < 0.01), whereas no significant differences were observed between the 11.5% CP (CLPD) group and any other groups for these amino acids (*p* > 0.05). Other amino acids, ASP, Thr, Ser, Glu, Gly, Ala, Val, Met, Ile, Leu, Lys, His, Arg, and Pro, exhibited no significant differences across all treatment groups (*p* > 0.05). The levels of Phe and Tyr in the leg muscle exhibited a significant linear relationship with dietary crude protein levels (P_linear < 0.05), indicating an upward trend in the deposition of Phe and Tyr amino acids in the leg muscle as CP levels were reduced in the CLPD groups.

### 4.8. Intestinal Morphology Parameters

**Figure 1:**
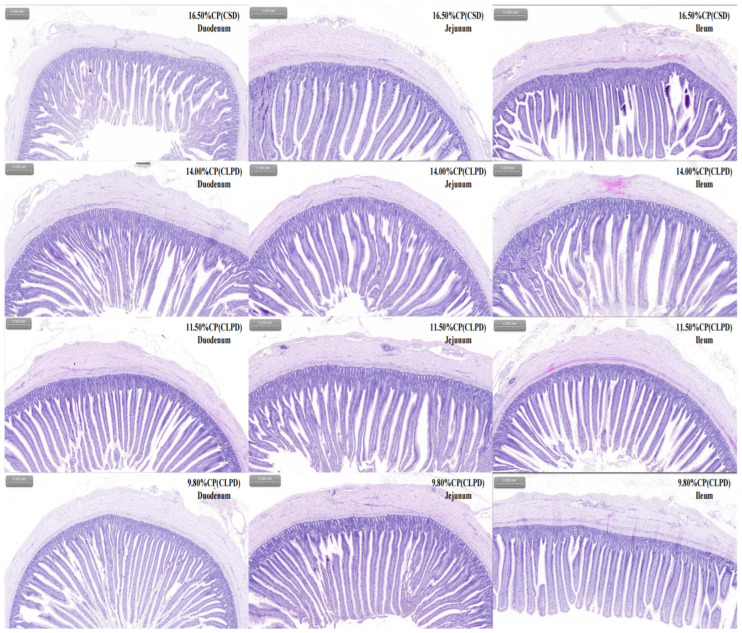
Effects of low-protein compound amino acid balanced diets on intestinal histological observation of Sanhua geese (100×).

As shown in Figure 1 and Table 9, the effects of dietary treatments on intestinal morphology parameters in geese varied by intestinal segment. In the duodenum, villus height was significantly greater in the 11.5% CP (CLPD) and 9.8% CP (CLPD) groups compared to the 16.5% CP (CSD) and 14.0% CP (CLPD) groups (*p* < 0.01). Crypt depth in the 9.8% CP (CLPD) group was significantly lower than in the 16.5% CP (CSD) group (*p* < 0.05), and both the villus-to-crypt ratio and muscularis mucosa thickness were significantly greater in the 9.8% CP (CLPD) group than in the 16.5% CP (CSD) group (*p* < 0.01). In the jejunum, villus height in the 16.5% CP (CSD) group was significantly lower than in the 14.0% CP (CLPD), 11.5% CP (CLPD), and 9.8% CP (CLPD) groups (*p* < 0.01). Crypt depth in the 16.5% CP (CSD) group was lower than in the 14.0% CP (CLPD) group (*p* < 0.05), and muscularis mucosa thickness showed a significant gradient of 16.5% CP (CSD) > 14.0% CP (CLPD) > 9.8% CP (CLPD) (*p* < 0.01). In the ileum, only the 14.0% CP (CLPD) group exhibited significantly lower muscularis mucosa thickness compared to the 16.5% CP (CSD), 11.5% CP (CLPD), and 9.8% CP (CLPD) groups (*p* < 0.01). No significant differences were observed in other intestinal morphology parameters across all segments (*p* > 0.05). The CP levels exhibited significant linear effects on VH and the VH/CD ratio in the duodenum (P_linear < 0.01), as well as on MMLT in the duodenum and jejunum (P_linear < 0.01), indicating improved intestinal structure in the CLPD groups.

### 4.9. Alpha Diversity of Cecal Contents and Microbial Community Structure Analysis

**Figure 2:**
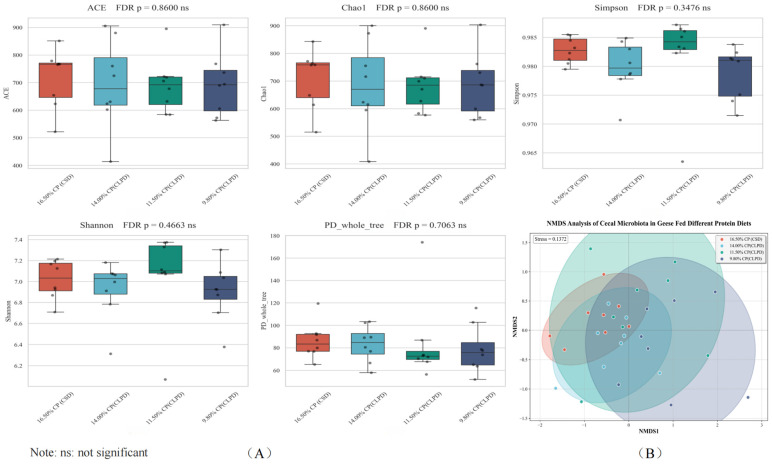
(**A**): Effects of low-protein compound amino acid balanced diets on alpha diversity of cecal contents in Sanhua Geese. (**B**): Effects of low-protein compound amino acid-balanced diets on PCoA Analysis of cecal contents in meat geese (n = 8 samples per group).

The effects of different dietary treatments on the alpha diversity of cecal contents in geese are shown in Figure 2A. There were no significant differences in ACE, Chao1, Simpson, Shannon, and PD_whole_tree among the treatment groups (*p* > 0.05). The non-metric multidimensional scaling (NMDS) analysis of cecal microbiota in geese fed diets with different CP levels is shown in Figure 2B, and the stress value was 0.1372 (falling within the range of 0.1–0.2, which indicates an acceptable fit for reflecting differences in microbial communities). The NMDS plot revealed partial clustering patterns of microbial communities among the four groups: samples from the 9.8% CP (CLPD) group tended to cluster in the right region along the NMDS1 axis; samples from the 16.5% CP (CSD) group were more concentrated in the left region; while samples from the 14.0% CP (CLPD) and 11.5% CP (CLPD) groups showed relatively dispersed distributions and overlapped with other groups to varying degrees. These patterns suggested that dietary CP levels potentially contributed to differences in cecal microbial composition, although there was no complete distinct separation among all groups due to partial overlap.

### 4.10. Taxonomic Composition and LEfSe Analysis of Cecal Microbiota

**Figure 3:**
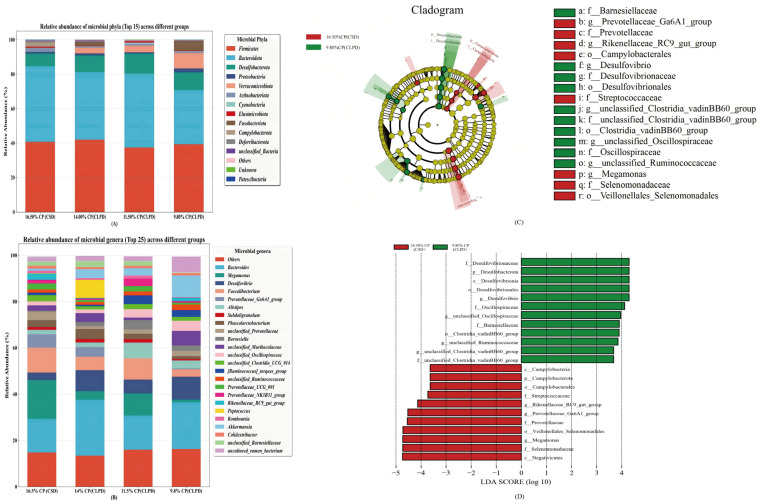
Effects of low-protein compound amino acid-balanced diets on the abundance and taxonomic structure of cecal microbiota in Sanhua geese (n = 8 samples per group). (**A**): Relative abundance of microbial phyla (Top 15) across different groups; (**B**): Relative abundance of microbial genera (Top 25) across different groups; (**C**): LEfSe cladogram showing differential abundance of microbial taxa between groups; (**D**): LDA score plot of differentially abundant microbial taxa between groups.

The structure and abundance of the cecal microbiota, influenced by the SBM reduction strategy, are shown in Figure 3A,B. At the phylum level (Figure 3A), the microbiota composition remained similar across all treatment groups, with the most abundant phyla being Firmicutes, Bacteroidota, Desulfobacterota, Proteobacteria, Verrucomicrobiota, Actinobacteriota, Cyanobacteria, Elusimicrobiota, Fusobacteriota, and Campylobacterota. At the genus level (Figure 3B), the most prevalent genera included *Bacteroides*, *Megamonas*, *Desulfovibrio*, *Faecalibacterium*, and *Prevotellaceae_Ga6A1_group*, among others. LEfSe analysis (Figure 3C,D) identified *Megamonas*, *Prevotellaceae_Ga6A1_group*, and *Rikenellaceae_RC9_gut_group* as significant biomarkers for the 16.5% CP (CSD) group (LDA scores > 4.0), while *Desulfovibrio*, *unclassified_Ruminococcaceae*, *unclassified_Oscillospiraceae* were identified as key biomarkers for the 9.8% CP (CLPD) group (LDA scores > 3.0). These findings suggest distinct shifts in microbiota composition related to dietary treatments, with potential implications for metabolic processes.

### 4.11. Dietary Effects on Cecal Microbiota at Phylum and Genus Levels

**Figure 4:**
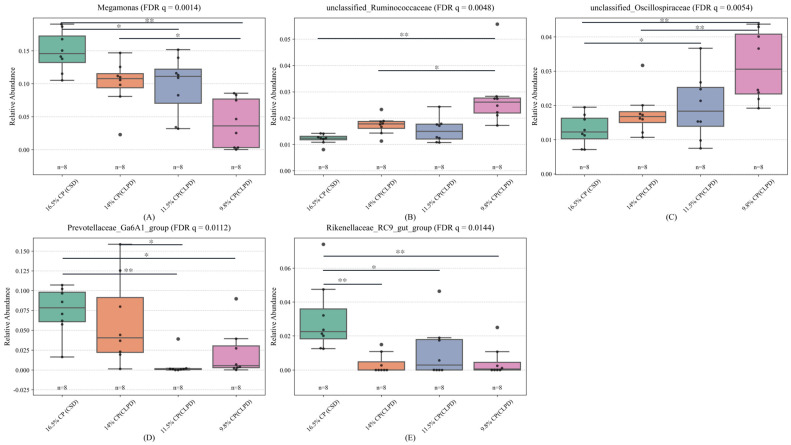
Effects of low-protein compound amino acid-balanced diets on the abundance of different microbial genera in the Cecum of Sanhua Geese. (**A**): Relative abundance of the *Megamonas* genus in the 16.5% CP (CSD), 14.0% CP (CLPD), 11.5% CP (CLPD), and 9.8% CP (CLPD) groups. (**B**): Relative abundance of the *unclassified_Ruminococcaceae* genus in the 16.5% CP (CSD), 14.0% CP (CLPD), 11.5% CP (CLPD), and 9.8% CP (CLPD) groups. (**C**): Relative abundance of the *unclassified_Oscillospiraceae* genus in the 16.5% CP (CSD), 14.0% CP (CLPD), 11.5% CP (CLPD), and 9.8% CP (CLPD) groups. (**D**): Relative abundance of the *Prevotellaceae_Ga6A1_group* genus in the 16.5% CP (CSD), 14.0% CP (CLPD), 11.5% CP (CLPD), and 9.8% CP (CLPD) groups. (**E**): Relative abundance of the *Rikenellaceae_RC9_gut_group* genus in the 16.5% CP (CSD), 14.0% CP (CLPD), 11.5% CP (CLPD), and 9.8% CP (CLPD) groups. Note: Asterisks (*) indicate statistically significant differences between groups. *p* < 0.05 is indicated by *, *p* < 0.01 is indicated by **.

To further validate these differences, the relative abundances of the identified taxa were analyzed using one-way analysis of variance (ANOVA), followed by Tukey′s multiple comparison test, with *p*-values adjusted via the false discovery rate (FDR) method to account for multiple comparisons (Figure 4). The results, based on adjusted *p*-values (*p*), are as follows: (1) The relative abundance of *Megamonas* (Figure 4A) was significantly higher in the 16.5% CP (CSD) group compared to the 11.5% CP (CLPD) and 9.8% CP (CLPD) groups (*p* < 0.01), and in the 14.0% CP (CLPD) group compared to the 9.8% CP (CLPD) group (*p* < 0.05), while no significant differences were observed between the 14.0% CP (CLPD) group and the 11.5% CP (CLPD) or 9.8% CP (CLPD) groups (*p* > 0.05); (2) The relative abundance of *unclassified_Ruminococcaceae* (Figure 4B) was significantly higher in the 9.8% CP (CLPD) group compared to the 16.5% CP (CSD) and 14.0% CP (CLPD) groups (*p* < 0.05), with no significant differences among the remaining groups (*p* > 0.05); (3) The relative abundance of *unclassified_Oscillospiraceae* (Figure 4C) was significantly higher in the 9.8% CP (CLPD) group compared to the 16.5% CP (CSD) and 14.0% CP (CLPD) groups (*p* < 0.05), the 11.5% CP (CLPD) group had a significantly higher than the 16.5% CP (CLPD) group (*p* < 0.05), while there was no significant difference between the 16.5% CP (CLPD) group and the 14.00% CP (CLPD) group(*p* > 0.05); (4) The relative abundance of *Prevotellaceae_Ga6A1_group* (Figure 4D) was significantly higher in the 16.5% CP (CSD) group compared to the 11.5% CP (CLPD) and 9.8% CP (CLPD) groups (*p* < 0.05), and in the 14.0% CP (CLPD) group compared to the 11.5% CP (CLPD) group (*p* < 0.05), while no significant difference was observed between the 11.5% CP (CLPD) and 9.8% CP (CLPD) groups (*p* > 0.05); (5) The relative abundance of *Rikenellaceae_RC9_gut_group* (Figure 4E) was significantly higher in the 16.5% CP (CSD) group compared to the other groups (*p* < 0.05), with no significant differences among the remaining groups (*p* > 0.05).

### 4.12. Correlation of Cecal Genera with Geese Morphology and Physiology

**Figure 5:**
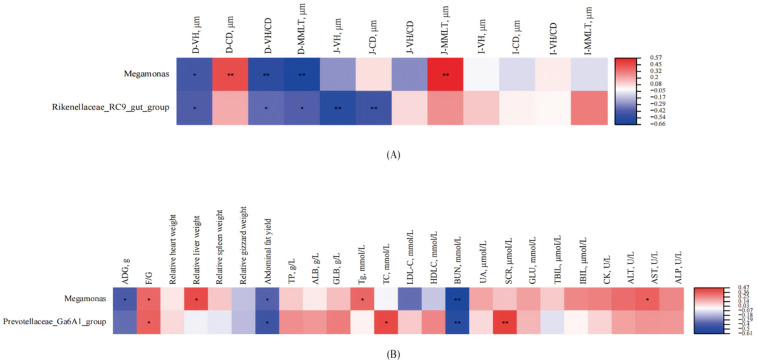
Effects of low-protein compound amino acid balanced diets on spearman correlation analysis between cecal genera and growth performance, organ weights, serum biochemical parameters, and intestinal morphology in Sanhua geese. (**A**): Spearman Correlation Analysis Between Cecal Genera and Intestinal Morphology. (**B**): Spearman Correlation Analysis Between Cecal Genera and Growth Performance, Serum Biochemical Parameters. Note: D-VH: Duodenal villus height, D-CD: Duodenal crypt depth, D-VH/CD: Duodenal villus to crypt ratio, D-MMLT: Duodenal mucosal muscle layer thickness, J-VH: Jejunal villus height, J-CD: Jejunal crypt depth, J-VH/CD: Jejunal villus to crypt ratio, J-MMLT: Jejunal mucosal muscle layer thickness, I-VH: Ileal villus height, I-CD: Ileal crypt depth, I-VH/CD: Ileal villus to crypt ratio, I-MMLT: Ileal mucosal muscle layer thickness. Asterisks (*) indicate statistically significant differences between groups; *p* < 0.05 is indicated by *, *p* < 0.01 is indicated by **.

As shown in Figure 5, significant correlations were observed between differential cecal genera and intestinal morphology, growth performance, and serum biochemical parameters in meat geese. Specifically, Figure 5A illustrates the correlations with intestinal morphological indices: *Megamonas* was significantly negatively correlated with duodenal villus height (VH, *p* < 0.05), villus height to crypt depth ratio (VH/CD, *p* < 0.05), and mucosal muscle layer thickness (MMLT, *p* < 0.05), but positively correlated with duodenal crypt depth (CD, *p* < 0.05) and jejunal MMLT (*p* < 0.05). Similarly, *Rikenellaceae_RC9_gut_group* exhibited significant negative correlations with duodenal VH (*p* < 0.05), VH/CD (*p* < 0.05), and MMLT (*p* < 0.05), as well as jejunal VH (*p* < 0.05) and CD (*p* < 0.05). Figure 5B presents the correlations with growth performance and serum biochemical parameters: *Megamonas* showed significant negative correlations with average daily gain (ADG, *p* < 0.05), while displaying positive correlations with feed-to-gain ratio (F/G, *p* < 0.05), relative liver weight (*p* < 0.05), and serum triglyceride (TG, *p* < 0.05) levels. Additionally, *Prevotellaceae_Ga6A1_group* was positively correlated with F/G (*p* < 0.05), serum total cholesterol (TC, *p* < 0.05), and creatinine (SCR, *p* < 0.05), but negatively correlated with abdominal fat percentage (*p* < 0.05).

## 5. Discussion

To mitigate the challenges posed by the meat goose industry’s heavy reliance on SBM—including raw material price volatility, geopolitical risks, and environmental pressures—this study developed and evaluated a sustainable low-protein compound diet system. This approach entailed a progressive reduction in dietary SBM content, supplemented by alternative feedstuffs such as rapeseed meal, corn distillers DDGS, broken rice, and rice bran, with dietary formulations optimized through restrictive amino acid balancing. Results revealed that this low-protein feeding strategy significantly enhanced the growth performance of meat geese. Specifically, geese in the CLPD groups (B, C, and D) exhibited a markedly higher ADG and improved feed conversion ratio compared to the control group. These findings suggest that, by refining dietary composition and ensuring amino acid equilibrium, CLPD can sustain or even augment growth outcomes in meat geese while substantially improving nitrogen utilization efficiency. This strategy offers a practical avenue for reducing SBM dependency, providing valuable implications for the sustainable advancement of poultry production.

The observed improvements in growth performance are intricately linked to a suite of physiological and metabolic adaptations. As dietary protein levels declined, the apparent digestibility of crude protein and multiple amino acids in geese increased progressively. This enhancement is likely attributable to the elevated bioavailability of synthetic amino acids, which, existing in free form, are readily absorbed by the intestine without requiring extensive enzymatic degradation [24]. Such rapid uptake accelerates protein turnover, ensuring an adequate amino acid supply for muscle development. Additionally, the diminished inclusion of SBM likely alleviated the adverse effects of trypsin inhibitors inherent in SBM [25], thereby enhancing endogenous protease activity and boosting overall feed digestibility. A notable shift in the dietary energy-to-protein ratio prompted a reallocation of metabolic resources. Protein synthesis, encompassing transcription, translation, and folding, demands considerable ATP, whereas lipid synthesis is comparatively energy-efficient. Consequently, CLPD redirected energy toward rapid fat deposition rather than muscle protein accretion [26], accounting for the accelerated body weight gain and elevated abdominal fat proportion in geese fed these diets. Increased serum low-density lipoprotein cholesterol levels, which may indicate heightened cholesterol transport demands, could further support this potential metabolic shift [27]. Although we balanced the limiting amino acids (lysine, methionine, and threonine), previous studies suggest that a further reduction in dietary protein levels (below 10%) might lead to relative deficiencies in certain essential amino acids, such as leucine and arginine. These potential deficiencies could plausibly impair the efficiency of ribosomal protein synthesis downstream of the mTORC1 signaling pathway, potentially reducing the overall efficiency of protein synthesis in the organism, as reported in prior research [28]. As a result, unutilized amino acids are metabolized and released into the bloodstream, leading to elevated blood amino acid concentrations and a concurrent decrease in amino acid deposition in muscle tissue. This observation is consistent with the primary findings of our experimental study.

At the muscular level, responses to CLPD displayed distinct site-specific patterns. In breast muscle, concentrations of glycine, alanine, and aromatic amino acids were significantly diminished. Concurrently, rapid fat synthesis and deposition, coupled with sustained insulin elevation, may induce “anabolic resistance” in fast-twitch fibers, potentially diminishing mTORC1 pathway responsiveness to amino acids and channeling carbon skeletons toward adipose tissue, as hypothesized in previous literature [29]. This mechanism elucidates the heightened sensitivity of breast muscle to LPD. Conversely, leg muscle, dominated by slow-twitch fibers, exhibited greater metabolic stability, with elevated tyrosine and phenylalanine levels detected only in the group with the most substantial SBM reduction (group D). This anomaly likely stems from insufficient phenylalanine intake due to lower SBM levels, constraining tetrahydrobiopterin synthesis—a critical cofactor for phenylalanine hydroxylase—and impeding phenylalanine conversion to tyrosine [30]. These differential responses highlight the potential of targeted supplementation with branched-chain and methyl-donor amino acids, alongside comprehensive optimization of the dietary amino acid profile, to enhance breast muscle protein synthesis efficiency and maintain leg muscle aromatic amino acid homeostasis while reducing SBM reliance, ultimately preserving meat quality.

The influence of CLPD on intestinal morphology also exhibited pronounced segment-specificity, predominantly affecting the duodenum and jejunum, with minimal impact on the ileum. In the duodenum, groups C and D displayed significantly greater villus height and reduced crypt depth relative to groups A and B, culminating in an elevated V/C in group D—a trait closely associated with improved nutrient absorption [31]. Additionally, the thickened mucosal muscularis in group D likely bolstered intestinal mechanical strength and peristalsis [32]. In the jejunum, CLPD similarly fostered villus elongation, yet mucosal muscularis thickness declined with decreasing protein levels, possibly reflecting adaptive responses to altered mechanical demands, the mechanisms of which merit further exploration. By contrast, ileal morphology remained largely unaltered, with only minor shifts in muscularis thickness in select groups, consistent with its primary role in water and electrolyte absorption [33]. Remarkably, despite progressive SBM reduction, intestinal morphology showed no detrimental effects; instead, enhancements in duodenal and jejunal parameters emerged. This improvement likely arises from the combined reduction in SBM-derived antinutritional factors and optimized amino acid provision via synthetic supplements, synergistically enhancing nutrient absorption and feed efficiency in the proximal intestine.

Using 16S rRNA sequencing, this study assessed the effects of CLPD on cecal microbiota, elucidating how SBM reduction modulates gut microbial communities to bolster growth and metabolic efficiency. Dietary treatments exerted no significant influence on cecal microbial alpha diversity, corroborating prior evidence that moderate protein reductions preserve microbial richness and stability [34]. However, beta diversity analyses revealed distinct microbial community structures across groups, with the greatest divergence between the 16.5%CP (CSD) and the 9.8%CP (CLPD) groups, indicating that substantial protein level alterations drive microbial restructuring. *Megamonas*, potentially linked to fat deposition and energy metabolism, declined in abundance under the high energy-to-protein ratio of CLPD, consistent with observations in laying hens on high-energy diets [35]. Previous studies have shown that the improvement of cecal microflora in geese is accompanied by a significant increase in the relative abundance of *Oscillospiraceae* and *Ruminococcaceae*. As important producers of short-chain fatty acids (SCFAs) in the intestine [36,37], the butyric acid produced by *Oscillospiraceae* and *Ruminococcaceae* helps to directly support the function of intestinal epithelial cells and maintain the intestinal barrier function [38]. This study found that a low-protein, diversified diet is conducive to the colonization of these bacteria in the cecum of geese, promoting their decomposition of cellulose in the diet and improving the feed conversion efficiency. *Prevotellaceae_Ga6A1_group*, implicated in cellulose degradation for high-fiber digestion [39], decreased possibly due to DDGS inclusion, mirroring findings from brewer’s grain supplementation in Landes geese [37]. *Rikenellaceae_RC9_gut_group*, engaged in bile acid, protein, lipid, and carbohydrate metabolism [40], diminished with reduced crude fiber and increased energy feed proportions in LPD [41]. In contrast, *Megamonas* positively correlated with F/G and BUN and negatively with ADG, abdominal fat, and intestinal morphology, implying nitrogen wastage and intestinal suppression in high-protein contexts [42]. *Prevotellaceae_Ga6A1_group* positively correlated with serum cholesterol and creatinine, hinting at lipid and renal regulatory roles, while *Rikenellaceae_RC9_gut_group* negatively correlated with intestinal morphology, reinforcing high-protein diets’ inhibitory effects. Collectively, CLPD with amino acid balancing selectively modulates cecal microbiota, enhancing growth, intestinal health, and nitrogen efficiency, thus supporting sustainable poultry production.

## 6. Conclusions

This study finds that a low-protein compound diet with precisely balanced limiting amino acids can reduce SBM usage, improve feed utilization, and enhance meat geese’s growth performance, protein efficiency, and intestinal development by regulating nitrogen metabolism and lipid deposition. However, crude protein levels below 10% cause amino acid imbalance and adverse metabolic effects, while 11.5% with balanced amino acids optimizes growth and homeostasis.

## Figures and Tables

**Table 1:** Ingredients and nutrient compositions of experimental diets (air dry basis).

Items	CP Level (%)			
	16.5% (CSD)	14.0% (CLPD)	11.5% (CLPD)	9.8% (CLPD)
Ingredient, %				
Maize	67.33	49.81	53.76	57.60
Soybean meal (43% CP)	22.29	9.20	5.00	2.53
Rice Husk	3.17	3.63	4.95	6.86
Wheat	/	8.21	8.83	4.50
Wheat bran	/	1.20	2.93	1.31
Rice bran	/	1.20	1.22	2.96
Broken rice	/	8.50	5.57	9.57
Rapeseed meal	/	3.00	3.00	3.00
DDGS	/	6.50	4.88	1.41
L-Lysine	0.17	0.55	0.72	0.83
DL-Methionine	0.06	0.14	0.21	0.28
L-Threonine	0.12	0.27	0.36	0.42
L-Valine	0.09	0.16	0.27	0.36
Limestone Powder	0.84	0.90	0.90	0.89
Dicalcium Phosphate	1.46	1.34	1.39	1.48
^2^ Premix	3.00	3.00	3.00	3.00
Soybean oil	1.07	1.99	2.61	2.60
Salt	0.40	0.40	0.40	0.40
^1^ Nutritional leve1				
Metabolizable Energy, MJ/kg	11.10	11.09	11.09	11.09
Crude Protein, %	16.50 (16.72)	14.00 (14.21)	11.50 (11.37)	9.80 (9.87)
Crude Fibre, %	5.00	5.00	5.00	5.00
Ether Extract, %	5.50	5.50	5.50	5.50
Lysine, %	0.90 (0.94)	0.90 (0.91)	0.90 (0.96)	0.90 (0.95)
Methionine + Cysteine, %	0.67 (0.70)	0.67 (0.61)	0.67 (0.62)	0.67 (0.62)
Threonine, %	0.58 (0.60)	0.58 (0.61)	0.58 (0.63)	0.58 (0.56)
Valine, %	0.70 (0.74)	0.70 (0.73)	0.70 (0.71)	0.70 (0.67)
Tryptophan, %	0.21	0.21	0.21	0.21
Calcium, %	0.80	0.80	0.80	0.80
Non-phytate Phosphorus, %	0.37	0.37	0.37	0.38
Acid-Insoluble Ash	0.30	1.50	1.20	1.70
Cost, USD/ton	354.5	341.38	333.57	328.83

^1^ Nutrient concentration: Nutritional requirements are based on the Chinese agricultural industry standards “Nutritional Requirements for Geese” (NY/T 4641-2025) and “Feeding Standards for Commercial Meat Geese” (DB37/T 2784-2016). Values for crude protein (CP) and individual amino acids are presented as actual measured values (in parentheses), while values for the remaining nutritional indices are calculated values. Diet descriptions: 16.5% CP (CSD): Corn-soybean meal type control, primarily based on maize and soybean meal (16.5% crude protein level). 14.0% CP (CLPD): Composite low-protein diet type, with diversified ingredients such as rapeseed meal, DDGS, broken rice, wheat bran, rice bran, and wheat (14.0% crude protein level). 11.5% CP (CLPD): Composite low-protein diet type, similar diversified formulation as above (11.5% crude protein level). 9.8% CP (CLPD): Composite low-protein diet type, similar to a diversified formulation with gradient soybean meal replacement and crystalline amino acid supplementation (e.g., L-lysine, DL-methionine, L-threonine, L-valine) to match the CSD amino acid profile (9.8% crude protein level). ^2^ The premix provides per kilogram of feed: Vitamin A 7500 IU, Vitamin B1 1.0 mg, Vitamin B2 6 mg, Pantothenic acid 9.2 mg, Vitamin B6 1.8 mg, Vitamin B12 0.10 mg, Vitamin D3 1600 IU, Vitamin E 13.5 IU, Vitamin K3 2 mg, Biotin 0.1 mg, Folic acid 0.4 mg, Niacin 60 mg, Choline 1400 mg, Copper 6 mg, Iron 80 mg, Manganese 100 mg, Zinc 80 mg, Iodine 0.42 mg, Selenium 0.3 mg, Calcium 3 g, Phosphorus 0.99 g.

**Table 2:** Effects of low-protein compound amino acid balanced diets on the growth performance of Sanhua geese (means ± SD; n = 6 replicates/pens per group, 8 geese per replicate).

Item	Diet Treatment				P_Linear	FDR_Corrected_P
	16.5% (CSD)	14.0% (CLPD)	11.5% (CLPD)	9.8% (CLPD)		
IBW, g	1725.59 ± 189.41	1703.08 ± 179.67	1714.81 ± 226.65	1725.79 ± 184.23	0.9950	0.9249
FBW, g	3902.20 ± 407.62 ^B^	4086.85 ± 487.36 ^AB^	4246.29 ± 488.75 ^A^	4203.90 ± 537.07 ^A^	0.0001	0.0013
ADG, g	62.19 ± 9.43 ^A^	68.49 ± 11.07 ^B^	72.33 ± 10.46 ^B^	70.80 ± 12.66 ^B^	*p* < 0.0001	0.0001
F/G	5.16 ± 0.34 ^A^	4.65 ± 0.27 ^B^	4.46 ± 0.31 ^B^	4.43 ± 0.34 ^B^	*p* < 0.0000	*p* < 0.0000
ADFI, g	320.07 ± 9.43	316.89 ± 4.88	321.77 ± 9.43	313.55 ± 13.41	0.5450	0.4777

Notes: IBW: Initial body weights; FBW: Final body weights; ADG: Average daily weight gain; ADFI: Average daily feed intake; F/G: Feed-to-gain ratio; ^A,B^ Means within a row with different superscript letters differ highly significantly (*p* < 0.01).

**Table 3:** Effects of low-protein compound amino acid balanced diets on relative organ weights of Sanhua geese (means ± SD, n = 8 samples per group).

Items	Diet Treatment				P_Linear	FDR_Corrected_P
	16.5% (CSD)	14.0% (CLPD)	11.5% (CLPD)	9.8% (CLPD)		
Heart yield, %	0.73 ± 0.07	0.74 ± 0.08	0.75 ± 0.06	0.72 ± 0.07	0.8726	0.7776
Liver yield, %	2.26 ± 0.25 ^a^	2.12 ± 0.34 ^a^	2.40 ± 0.34 ^a^	1.94 ± 0.21 ^b^	0.5822	0.0471
Spleen yield, %	0.09 ± 0.02	0.11 ± 0.04	0.11 ± 0.04	0.10 ± 0.03	0.8656	0.7776
Gizzard yield, %	3.67 ± 0.46	3.62 ± 0.46	3.96 ± 0.30	3.74 ± 0.27	0.5822	0.5308
Abdominal fat percentage yield, %	1.46 ± 0.50 ^B^	1.32 ± 0.47 ^B^	2.55 ± 0.67 ^A^	2.58 ± 0.64 ^A^	*p* < 0.0001	0.0003

^a,b^ Means with different superscript letters in the same row differ significantly (*p* < 0.05). ^A,B^ Means within a row with different superscript letters differ highly significantly (*p* < 0.01). The same applies to the following table.

**Table 4:** Effects of low-protein compound amino acid-balanced diets on apparent digestibility of Sanhua geese (means ± SD, n = 6 samples per group).

Items	Diet Treatment				P_Linear	FDR_Corrected_P
	16.5% (CSD)	14.0% (CLPD)	11.5% (CLPD)	9.8% (CLPD)		
CP, %	41.54 ± 8.47 ^C^	40.37 ± 2.58 ^C^	54.80 ± 11.54 ^B^	66.01 ± 2.08 ^A^	*p* < 0.0000	*p* < 0.0000
ASP, %	80.02 ± 2.14 ^C^	80.37 ± 0.82 ^C^	83.93 ± 2.18 ^B^	87.64 ± 0.65 ^A^	*p* < 0.0000	*p* < 0.0000
Thr, %	77.07 ± 2.09 ^C^	86.98 ± 0.52 ^B^	89.79 ± 0.98 ^A^	88.80 ± 0.33 ^A^	*p* < 0.0000	*p* < 0.0000
Ser, %	81.18 ± 1.47 ^C^	80.63 ± 0.98 ^C^	84.71 ± 2.31 ^B^	86.63 ± 0.75 ^A^	0.0200	*p* < 0.0000
Glu, %	86.27 ± 1.30 ^D^	88.88 ± 0.39 ^B^	84.09 ± 1.23 ^C^	92.95 ± 0.40 ^A^	0.0001	*p* < 0.0000
Gly, %	48.70 ± 6.37 ^B^	42.44 ± 12.32 ^B^	70.39 ± 3.38 ^A^	67.98 ± 6.41 ^A^	*p* < 0.0000	*p* < 0.0000
Ala, %	74.11 ± 3.42 ^C^	78.44 ± 2.97 ^B^	79.87 ± 1.29 ^B^	89.14 ± 0.47 ^A^	*p* < 0.0000	*p* < 0.0000
Val, %	79.49 ± 1.49 ^D^	86.38 ± 0.73 ^B^	83.33 ± 1.31 ^C^	91.99 ± 0.31 ^A^	*p* < 0.0000	*p* < 0.0000
Ile, %	78.55 ± 1.87 ^D^	80.14 ± 1.00 ^C^	85.04 ± 0.61 ^B^	87.51 ± 0.51 ^A^	*p* < 0.0000	*p* < 0.0000
Leu, %	84.04 ± 1.31 ^D^	85.74 ± 0.60 ^C^	88.53 ± 0.74 ^B^	91.35 ± 0.32 ^A^	*p* < 0.0000	*p* < 0.0000
Tyr, %	80.11 ± 3.10 ^BC^	78.74 ± 2.15 ^C^	82.26 ± 1.25 ^B^	89.58 ± 1.04 ^A^	*p* < 0.0000	*p* < 0.0000
Phe, %	81.81 ± 1.50 ^D^	84.53 ± 0.79 ^C^	86.73 ± 1.12 ^B^	90.52 ± 0.61 ^A^	*p* < 0.0000	*p* < 0.0000
Lys, %	83.36 ± 1.26 ^D^	89.52 ± 0.59 ^B^	88.05 ± 1.12 ^C^	95.02 ± 0.41 ^A^	0.0013	*p* < 0.0000
His, %	85.05 ± 1.78 ^B^	85.06 ± 0.97 ^B^	84.73 ± 1.14 ^B^	90.03 ± 0.34 ^A^	0.0005	*p* < 0.0000
Arg, %	84.59 ± 4.44 ^B^	85.49 ± 3.09 ^B^	86.97 ± 1.41 ^B^	92.09 ± 1.11 ^A^	0.0002	0.0010
Pro, %	83.81 ± 1.08 ^B^	84.74 ± 0.90 ^B^	83.86 ± 0.52 ^B^	89.02 ± 0.59 ^A^	*p* < 0.0000	*p* < 0.0000

Notes: ^A,B,C,D^ Means within a row with different superscript letters differ highly significantly (*p* < 0.01). CP: Crude Protein. ASP: Aspartic Acid, Thr: Threonine, Ser: Serine, Glu: Glutamic Acid, Gly: Glycine, Ala: Alanine, Val: Valine, Ile: Isoleucine, Leu: Leucine, Tyr: Tyrosine, Phe: Phenylalanine, Lys: Lysine, His: Histidine, Arg: Arginine, Pro: Proline.

**Table 5:** Effects of low-protein compound amino acid balanced diets on serum biochemical parameters of meat geese (means ± SD, n = 8 samples per group).

Items	Diet Treatment				P_Linear	FDR_Corrected_P
	16.5% (CSD)	14.0% (CLPD)	11.5% (CLPD)	9.8% (CLPD)		
TP, g/L	37.04 ± 7.83	36.18 ± 5.95	33.10 ± 7.21	35.85 ± 6.64	0.8204	0.6282
ALB, g/L	15.64 ± 3.00	15.83 ± 2.46	14.34 ± 2.57	15.83 ± 2.79	0.9704	0.5707
GLB, g/L	21.40 ± 4.87	20.36 ± 3.52	18.76 ± 4.77	20.03 ± 4.04	0.8152	0.6282
TC, mmol/L	4.42 ± 0.72	4.51 ± 0.71	4.09 ± 0.49	4.64 ± 0.82	0.8204	0.4201
LDL-c, mmol/L	0.73 ± 0.16 ^B^	0.75 ± 0.23 ^B^	0.64 ± 0.12 ^B^	1.08 ± 0.30 ^A^	0.3429	0.0102
HDL-c, mmol/L	2.50 ± 0.49	2.75 ± 0.53	2.42 ± 0.43	2.78 ± 0.47	0.7268	0.4201
UA, μmol/L	185.29 ± 46.18	227.75 ± 59.92	214.71 ± 67.90	161.88 ± 37.90	0.7628	0.4201
SCR, μmoI/L	7.23 ± 3.38 ^A^	11.46 ± 4.52 ^A^	5.63 ± 2.71 ^B^	4.65 ± 1.82 ^B^	0.3852	0.0102
GLU, mmoI/L	11.32 ± 1.78	11.41 ± 1.60	10.86 ± 1.64	10.14 ± 1.62	0.6605	0.6282
TBIL, μmol/L	3.40 ± 1.05	3.55 ± 1.19	5.07 ± 1.62	4.32 ± 1.06	0.3429	0.4201
IBIL, μmol/L	3.18 ± 1.09	3.30 ± 1.29	4.74 ± 1.81	3.52 ± 1.12	0.6363	0.5780
ALT, U/L	14.67 ± 1.21	12.43 ± 4.65	13.86 ± 4.95	9.13 ± 4.49	0.5048	0.1201
AST, U/L	34.14 ± 3.48	36.00 ± 6.97	27.17 ± 4.92	25.63 ± 9.86	0.5048	0.4201
ALP, U/L	986.00 ± 144.03	986.63 ± 218.84	839.00 ± 143.66	858.71 ± 300.26	0.7268	0.8365

Note: TP: ^A,B^ Means within a row with different superscript letters differ highly significantly (*p* < 0.01). Total Protein, ALB: Albumin, GLB: Globulin, TC: Total Cholesterol, LDL-c: Low-Density Lipoprotein Cholesterol, HDL-c: High-Density Lipoprotein Cholesterol, UA: Uric Acid, SCR: Serum Creatinine, GLU: Glucose, TBIL: Total Bilirubin, IBIL: Indirect Bilirubin, ALT: Alanine Aminotransferase, AST: Aspartate Aminotransferase, ALP: Alkaline Phosphatase.

**Table 6:** Effects of low-protein compound amino acid balanced diets on serum amino acid composition of Sanhua geese (means ± SD, n = 8 samples per group).

Items	Diet Treatment				P_Linear	FDR_Corrected_P
	16.5% (CSD)	14.0% (CLPD)	11.5% (CLPD)	9.8% (CLPD)		
ASP, %	0.38 ± 0.06	0.38 ± 0.03	0.41 ± 0.05	0.44 ± 0.03	0.0273	0.1026
Thr, %	0.23 ± 0.04	0.22 ± 0.03	0.24 ± 0.03	0.25 ± 0.02	0.1216	0.3212
Ser, %	0.22 ± 0.04	0.22 ± 0.02	0.23 ± 0.03	0.25 ± 0.02	0.1216	0.2970
Glu, %	0.58 ± 0.08	0.57 ± 0.05	0.61 ± 0.07	0.66 ± 0.04	0.0273	0.1026
Gly, %	0.14 ± 0.03	0.14 ± 0.02	0.15 ± 0.02	0.16 ± 0.01	0.0601	0.1938
Ala, %	0.21 ± 0.03	0.20 ± 0.02	0.22 ± 0.03	0.23 ± 0.02	0.0601	0.1793
Val, %	0.23 ± 0.04	0.22 ± 0.02	0.24 ± 0.03	0.26 ± 0.02	0.0791	0.1938
Ile, %	0.16 ± 0.02	0.16 ± 0.01	0.17 ± 0.02	0.18 ± 0.01	0.0452	0.1294
Leu, %	0.32 ± 0.04	0.32 ± 0.03	0.35 ± 0.04	0.37 ± 0.03	0.0273	0.1026
Tyr, %	0.19 ± 0.02	0.19 ± 0.03	0.20 ± 0.03	0.22 ± 0.02	0.0791	0.1938
Phe, %	0.21 ± 0.03	0.20 ± 0.04	0.21 ± 0.02	0.23 ± 0.01	0.4346	0.5278
Lys, %	0.34 ± 0.04	0.34 ± 0.03	0.35 ± 0.04	0.38 ± 0.02	0.0516	0.1294
His, %	0.11 ± 0.01	0.11 ± 0.01	0.11 ± 0.01	0.12 ± 0.01	0.0547	0.1793
Arg, %	0.23 ± 0.03	0.22 ± 0.02	0.24 ± 0.03	0.26 ± 0.02	0.0273	0.1026
Pro, %	0.20 ± 0.03	0.19 ± 0.02	0.21 ± 0.03	0.23 ± 0.01	0.0516	0.1307

Notes: ASP: Aspartic Acid, Thr: Threonine, Ser: Serine, Glu: Glutamic Acid, Gly: Glycine, Ala: Alanine, Val: Valine, Ile: Isoleucine, Leu: Leucine, Tyr: Tyrosine, Phe: Phenylalanine, Lys: Lysine, His: Histidine, Arg: Arginine, Pro: Proline.

**Table 7:** Effects of low-protein compound amino acid balanced diets on amino acid composition of breast muscle in Sanhua geese (means ± SD, n = 8 samples per group).

Items	Diet Treatment				P_Linear	FDR_Corrected_P
	16.5% (CSD)	14.0% (CLPD)	11.5% (CLPD)	9.8% (CLPD)		
ASP, %	1.71 ± 0.19	1.70 ± 0.59	1.61 ± 0.05	1.65 ± 0.10	0.1123	0.2486
Thr, %	0.87 ± 0.09	0.86 ± 0.03	0.82 ± 0.02	0.82 ± 0.05	0.0455	0.1599
Ser, %	0.76 ± 0.07	0.75 ± 0.03	0.71 ± 0.02	0.72 ± 0.04	0.0240	0.1088
Glu, %	2.79 ± 0.27	2.77 ± 0.10	2.62 ± 0.07	2.68 ± 0.15	0.0586	0.1599
Gly, %	0.96 ± 0.06 ^A^	0.95 ± 0.04 ^A^	0.88 ± 0.05 ^B^	0.84 ± 0.02 ^B^	0.0000	0.0001
Ala, %	1.14 ± 0.09 ^A^	1.13 ± 0.04 ^A^	1.05 ± 0.02 ^B^	1.04 ± 0.06 ^B^	0.1841	0.0797
Val, %	0.94 ± 0.08	0.93 ± 0.03	0.88 ± 0.03	0.89 ± 0.05	0.0004	0.0031
Met, %	0.44 ± 0.06	0.49 ± 0.06	0.42 ± 0.07	0.41 ± 0.07	0.0173	0.0797
Ile, %	0.90 ± 0.09 ^A^	0.89 ± 0.03 ^A^	0.82 ± 0.06 ^B^	0.82 ± 0.07 ^B^	0.0011	0.0072
Leu, %	1.57 ± 0.15 ^A^	1.55 ± 0.06 ^A^	1.43 ± 0.05 ^B^	1.43 ± 0.10 ^B^	0.0010	0.0072
Tyr, %	0.68 ± 0.02 ^A^	0.67 ± 0.03 ^AB^	0.63 ± 0.06 ^BC^	0.60 ± 0.05 ^C^	0.0007	0.0072
Phe, %	0.87 ± 0.03 ^A^	0.83 ± 0.04 ^AB^	0.78 ± 0.12 ^BC^	0.74 ± 0.07 ^C^	0.0007	0.0087
Lys, %	1.66 ± 0.14 ^A^	1.63 ± 0.06 ^A^	1.51 ± 0.06 ^B^	1.51 ± 0.10 ^B^	0.0010	0.0072
His, %	0.55 ± 0.06 ^a^	0.55 ± 0.04 ^a^	0.48 ± 0.06 ^b^	0.49 ± 0.06 ^b^	0.0112	0.0306
Arg, %	1.25 ± 0.10 ^A^	1.24 ± 0.04 ^A^	1.12 ± 0.04 ^B^	1.10 ± 0.06 ^B^	0.0000	0.0002
Pro, %	0.78 ± 0.03 ^A^	0.76 ± 0.03 ^A^	0.73 ± 0.03 ^B^	0.72 ± 0.02 ^B^	0.0000	0.0011

Notes: ^A,B,C^ Means within a row with different superscript letters differ highly significantly (*p* < 0.01). ^a,b^ Means with different superscript letters in the same row differ significantly (*p* < 0.05). ASP: Aspartic Acid, Thr: Threonine, Ser: Serine, Glu: Glutamic Acid, Gly: Glycine, Ala: Alanine, Val: Valine, Met: Methionine, Ile: Isoleucine, Leu: Leucine, Tyr: Tyrosine, Phe: Phenylalanine, Lys: Lysine, His: Histidine, Arg: Arginine, Pro: Proline.

**Table 8:** Effects of low-protein compound amino acid balanced diets on amino acid composition of leg muscle in Sanhua geeses (means ± SD, n = 8 samples per group).

Items	Diet Treatment				P_Linear	FDR_Corrected_P
	16.5% (CSD)	14.0% (CLPD)	11.5% (CLPD)	9.8% (CLPD)		
ASP, %	1.78 ± 0.06	1.75 ± 0.13	1.78 ± 0.14	1.68 ± 0.07	0.5347	0.7604
Thr, %	0.89 ± 0.02	0.88 ± 0.06	0.90 ± 0.06	0.86 ± 0.03	0.6598	0.7604
Ser, %	0.77 ± 0.03	0.76 ± 0.05	0.78 ± 0.06	0.74 ± 0.03	0.6598	0.7604
Glu, %	2.91 ± 0.09	2.83 ± 0.18	2.92 ± 0.21	2.78 ± 0.09	0.5347	0.7604
Gly, %	0.81 ± 0.04	0.84 ± 0.06	0.83 ± 0.06	0.81 ± 0.03	0.9520	0.8045
Ala, %	1.10 ± 0.05	1.11 ± 0.07	1.12 ± 0.08	1.09 ± 0.03	0.7360	0.8568
Val, %	0.96 ± 0.04	0.95 ± 0.06	0.98 ± 0.07	0.95 ± 0.03	0.9351	0.8045
Met, %	0.52 ± 0.02	0.52 ± 0.04	0.55 ± 0.03	0.53 ± 0.02	0.9247	0.7603
Ile, %	0.91 ± 0.05	0.90 ± 0.06	0.94 ± 0.06	0.92 ± 0.02	0.7103	0.8045
Leu, %	1.58 ± 0.08	1.57 ± 0.10	1.62 ± 0.11	1.58 ± 0.04	0.8532	0.8045
Tyr, %	0.70 ± 0.05 ^B^	0.69 ± 0.04 ^B^	0.74 ± 0.05 ^AB^	0.79 ± 0.08 ^A^	0.0105	0.0267
Phe, %	0.84 ± 0.07 ^B^	0.84 ± 0.06 ^B^	0.90 ± 0.08 ^AB^	1.01 ± 0.13 ^B^	0.0105	0.0267
Lys, %	1.71 ± 0.09	1.68 ± 0.11	1.75 ± 0.11	1.73 ± 0.05	0.6598	0.8045
His, %	0.59 ± 0.05	0.60 ± 0.07	0.62 ± 0.05	0.61 ± 0.04	0.5347	0.8045
Arg, %	1.23 ± 0.08	1.24 ± 0.08	1.28 ± 0.07	1.28 ± 0.04	0.3142	0.7604
Pro, %	0.69 ± 0.02	0.69 ± 0.05	0.68 ± 0.05	0.66 ± 0.03	0.4204	0.7604

Notes: ^A,B^ Means within a row with different superscript letters differ highly significantly (*p* < 0.01). ASP: Aspartic Acid, Thr: Threonine, Ser: Serine, Glu: Glutamic Acid, Gly: Glycine, Ala: Alanine, Val: Valine, Met: Methionine, Ile: Isoleucine, Leu: Leucine, Tyr: Tyrosine, Phe: Phenylalanine, Lys: Lysine, His: Histidine, Arg: Arginine, Pro: Proline.

**Table 9:** Effects of low-protein compound amino acid balanced diets on intestinal morphological indices of Sanhua geese (means ± SD, n = 8 samples per group).

Item		Diet Treatment				P_Linear	FDR_Corrected_P
		16.5% (CSD)	14.0% (CLPD)	11.5% (CLPD)	9.8% (CLPD)		
Duodenum	VH, μm	1266.4225 ± 85.43 ^B^	1482.72 ± 145.97 ^B^	1776.44 ± 254.05 ^A^	1773.3686 ± 232.17 ^A^	0.0000	0.0004
	CD, μm	254.28 ± 51.81 ^a^	221.30 ± 51.52 ^ab^	231.34 ± 44.42 ^ab^	179.86 ± 26.20 ^b^	0.0078	0.0195
	VH/CD	5.17 ± 1.12 ^B^	7.20 ± 1.88 ^AB^	7.20 ± 1.93 ^AB^	10.01 ± 2.52 ^A^	0.0001	0.0005
	MMLT, μm	25.59 ± 2.22 ^B^	35.09 ± 6.71 ^AB^	32.44 ± 4.51 ^AB^	40.5 ± 5.75 ^A^	0.0001	*p* < 0.0000
Jejunum	VH, μm	1143.44 ± 40.97 ^B^	1675.87 ± 259.66 ^A^	1438.98 ± 71.02 ^A^	1453.67 ± 131.30 ^A^	0.1098	0.0001
	CD, μm	180.78 ± 31.14 ^B^	248.49 ± 54.49 ^A^	237.19 ± 56.35 ^AB^	184.92 ± 30.12 ^AB^	0.8000	0.0064
	VH/CD	6.54 ± 1.54	7.32 ± 2.80	6.23 ± 1.72	7.95 ± 0.82	0.4344	0.2776
	MMLT, μm	60.97 ± 9.66 ^A^	39.18 ± 8.32 ^B^	32.40 ± 8.31 ^BC^	24.48 ± 2.35 ^C^	*p* < 0.0000	*p* < 0.0000
Ileum	VH, μm	1260.53 ± 180.39	1273.22 ± 126.65	1155.59 ± 114.60	1220.93 ± 135.91	0.33566	0.3593
	CD, μm	192.78 ± 30.62	2227.25 ± 51.11	198.00 ± 30.44	191.19 ± 30.90	0.629675	0.3593
	VH/CD	6.67 ± 1.31	5.82 ± 1.43	5.91 ± 0.76	6.41 ± 1.08	0.629675	0.4200
	MMLT, μm	60.92 ± 14.38 ^A^	29.01 ± 5.32 ^B^	66.55 ± 22.00 ^A^	63.98 ± 9.87 ^A^	0.33566	0.0001

Notes: ^A,B,C^ Means within a row with different superscript letters differ highly significantly (*p* < 0.01). ^a,b^ Means with different superscript letters in the same row differ significantly (*p* < 0.05). VH: Villus Height, CD: Crypt Depth, VH/CD: Villus Height/Crypt Depth, MMLT: Mucosal Muscle Layer Thickness.

## Data Availability

The original contributions presented in this study are included in the article. Further inquiries can be directed to the corresponding author.
